# Integrated treatment vs. treatment-as-usual for recent onset schizophrenia; 12 year follow-up on a randomized controlled trial

**DOI:** 10.1186/1471-244X-13-200

**Published:** 2013-07-30

**Authors:** Víðir Sigrúnarson, Rolf W Gråwe, Gunnar Morken

**Affiliations:** 1 Department of Neuroscience, Norwegian University of Science and Technology, Trondheim, Norway; 2 Department of Psychiatry, St. Olav’s University Hospital, Trondheim, Norway; 3 Department of Research and Development, Drug and Alcohol Treatment Health Trust in Central Norway, Trondheim, Norway

**Keywords:** Schizophrenia, Integrated treatment, Early intervention, Long term outcome

## Abstract

**Background:**

The aim of this study is to compare the 12-year follow-up effects on in- and outpatient services of 2 years of integrated treatment for recent-onset schizophrenia versus treatment as usual in a randomized controlled trial.

**Methods:**

50 patients aged 18–35 years were randomized to Integrated Treatment (IT) (N = 30) or Treatment-as-Usual (TAU) (N = 20) for two years. TAU comprised optimal pharmacotherapy and outreach assertive treatment, while IT also included cognitive-behavioural family treatment, skills training, strategies for residual psychotic and non-psychotic problems and home-based crisis management.

**Results:**

There were no differences in number of days in hospital, time to readmission, number of admittances to psychiatric wards, number of involuntarily psychiatric admissions or number of outpatient contacts over a period of 12 years following the initial 2-year treatment trial. Fewer patients in the IT group were, however, involuntary admitted to hospital in the period.

**Conclusions:**

The intensive two-year psychosocial intervention seemed to have little long-term effects on use of in- and outpatient services.

**Trial registration:**

Current Controlled Trials: NCT00184509

## Background

Increased focus on the importance of early interventions in psychosis, with initiation of treatment during the “critical period”, has led to the development of time-limited specialized early interventions [1,2]. The aims of these early specialized treatments are to shorten the duration of untreated psychosis, preventing relapses and thereby improving prognosis [3,4].

There are three different early intervention approaches; 1) interventions focusing on recognizing and helping young individuals at high risk of developing psychosis (i.e., before debut of psychosis); 2) early identification programs where the goal is to identify and treat patients in the very beginning of the first psychotic episode, and 3) specialized services to patients with recent but established psychosis [1,5].

Randomized controlled trials (RCT) have shown promising effects of early specialized interventions on short term outcomes (reduction in hospital admissions and symptoms and improved function) or as long as the treatment continues [6-8]. Furthermore, early interventions in psychosis have been found to be cost effective compared to standard care [9].

The long-term effects of these treatments are, however, still unclear. In a review of outcomes at 3–5 years after early psychosis intervention, Bird et al. concluded that it is undetermined if the effects of early interventions are sustainable [2]. Except from indications of improved social outcomes, recent findings from 5-year follow-up studies have shown low effects on admission rate and mean number of bed days [10], level of psychotic and negative symptoms [11,12], global functioning, substance abuse, depression, and suicidal behaviour [12].

The aim of this study was to compare the long-term effects of 2 years of integrated treatment (IT) for recent-onset psychosis versus treatment as usual (TAU) in a randomized controlled trial. Firstly we hypothesized that integrated treatment would have long-term effects on number of days in hospital during the 12–year follow-up period. Hence, we also hypothesized that patients receiving integrated treatment would have reduced number of psychiatric admissions; number of outpatient psychiatric contacts, use of involuntary admissions, duration of inpatients and outpatient involuntary treatment, and mortality.

To our knowledge this is the first long-term RCT reporting a follow up on outcome 14 years after initiation of treatment.

## Methods

The present study was a centre in the International Optimal Treatment multi-site project initiated by Dr. Ian R.H. Falloon in 1994. The aim was to evaluate the effects of applying evidence-based integrated biomedical and psycho-social interventions in routine services for patients with recent-onset and chronic schizophrenia and other non-affective psychotic disorders [13]. The present study is based on the original RCT [6].

### Participants

From 1992 to 1997 at the St. Olav’s University Hospital in Trondheim, Norway, 50 consecutive patients with recent onset psychosis, referred to a specialized team for treatment of psychosis, were included in a RCT comparing IT and TAU. Recent onset was defined as having experienced their first symptoms of psychosis within the last two years before inclusion.

Clinically stable patients between 18–35 years, diagnosed with DSM-IV [14] schizophrenia, schizoaffective or schizophreniform disorders by a psychiatrist or a clinical psychologist trained to administer the Structured Clinical Interview for the DSM-IV, were invited to participate in the study. Patients with mental retardation, substance use disorders or who were not expected to reside in the county for at least one year after inclusion, were excluded. 30 patients were randomly allocated to two years of integrated psychosocial treatment (IT) and the remaining 20 were provided treatment-as-usual (TAU).

Prior to randomization to IT or TAU written informed consent was obtained and an independent assistant completed baseline assessments [6,15,16].

The mean age of the patients (30 men and 20 women) at admission was 25.4 (SD 4.6) years (Table 1), whereas the mean age at the end of follow-up was 39.0 (SD 4.6) years. At study entry the diagnoses (DSM-III-R; American Psychiatric Association, 1987) were: schizophrenia, 40 (80%); schizoaffective disorder, 6 (12%); and schizophreniform disorders, 4 (8%).

**Table 1:** Demographic and clinical characteristics for the integrated treatment (IT) and treatment as usual (TAU) groups at baseline

**Variables**	**All patients (n = 45)**	**IT group (n = 28)**	**TAU group (n = 17)**	**Statistics**	**P-value**
*Baseline demographics*					
Age. Mean (SD)	24.5 (4.5)	25.5 (4.8)	24.3 (4.2)	Independent t-test	.395
Gender. No. (%)					
Female	18 (40)	11 (39)	7 (41)	Chi-Square	.900
Male	27 (60)	17 (61)	10 (59)		
Hospitalized before study entry. No (%)					
Yes	39 (87)	27 (96)	12 (71)	Fishers Exact test	.023*
No	6 (13)	1 (4)	5 (29)		
Days hospitalized 12 months preceding study entry. Mean (SD)	131 (103)	131 (105)	131 (103)	Independent t-test	.983
*Psychiatric assessments, baseline.*					
Diagnoses (DSM-IV). No. (%)					
Schizophrenia	40 (80)	23 (76)	17 (85)		
Schizoaffective	6 (12)	5 (17)	1 (5)		
Schizophreniform	4 (8)	2 (7)	2 (10)		
GAF score. Mean (SD)	49.7 (10.7)	52.0 (11.5)	45.9 (8.3)	Independent t-test	.064
Total BPRS score. Mean (SD)	39.7 (7.6)	37.9 (7.5)	42.6 (7.2)	Independent t-test	.046*
Antipsychotic drugs, Chlorpromazine equivalent dose per day (mg). Mean (SD)	229 (118)	211 (93)	259 (148)	Independent t-test	.185

*significant difference.

The research was carried out in compliance with the Helsinki Declaration and the study was approved by The Regional Committee for Medical and Health Research Ethics in Central-Norway.

### Interventions

All participants received TAU which included regular out-patient case management with antipsychotic medication, crisis in-patient treatment when needed, supportive housing and day care, rehabilitation promoting work activities and independent living, supportive psychotherapy and brief psycho-education.

IT was based on methods described in published manuals [17,18] and was comparable to what was recommended as optimal treatment for schizophrenia in international guidelines at the time. In addition to TAU, IT patients were treated by a multi-disciplinary team independent of the TAU program. The caseload in the IT-team was low with patient staff ratio approximately 1 to 10. IT patients and their primary caregivers received cognitive–behavioural family communication and problem solving skills training, individual cognitive-behavioural strategies for residual symptoms and disability and structured single-family psycho-education. Education in use of medication and methods to improve medication adherence was given [13].

Most patients received weekly treatment-sessions during the first 2 months and then at least one treatment-session every third week during the first year. The second year of the project treatment-sessons were provided at least once each month. In periods of crisis and excercebations assertive out-reach crisis management was provided at home up to 3 hours per week, often supplemented with phone-consultations. For those patients who had limited contact with any informal caregivers, about 20%, family education and problem solving sessions were given as individual treatment-sessions. [6,15,16].

At the end of the 2 years treatment period participants from the IT group were transferred to treatment as usual.

### Outcomes

The primary follow-up outcome was the number of days in hospital during the 12 year follow-up period. Secondary objectives were number of psychiatric admissions, number of outpatient psychiatric contacts, use of involuntary admissions, duration of inpatient and outpatient involuntary treatment, and mortality.

### Randomization

**Figure 1:**
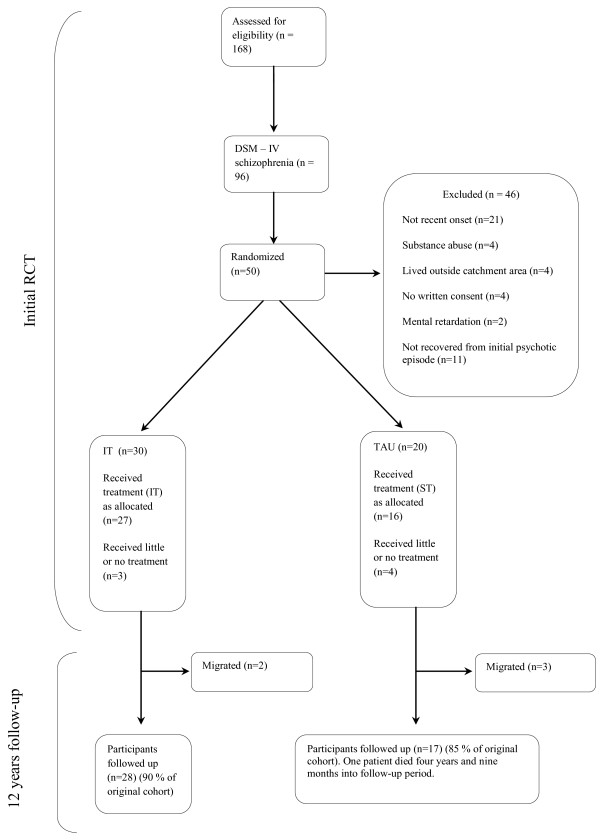
Participant flow.

Patients were randomly allocated to IT or TAU using a sequence of sealed pre-numbered envelopes with group assignments according to random numbers provided by the international Optimal Treatment Project administration. A secretary outside the clinical services opened the envelopes. Gender was stratified in blocks of varying size (between 8 and 12 and with a ratio of 3:2 of IT to TAU) ensuring that a majority of the patients received the experimental treatment [6]. Case recruitment, allocation and retention are summarized in Figure 1.

### Results from initial study

It has earlier been reported that IT was associated with greater reductions in negative symptoms, minor psychotic episodes, and in stabilising positive symptoms. The proportion of cases with excellent two-year clinical outcomes doubled, but 47% of cases remained in need of continuing treatment for their persisting symptoms and/or disability, or risk of recurrence. No effect on adherence to medication was found [6,15,16].

### Follow-up study

In 2011 and 2012 the participants of the study were assessed over a period of 12 years following completion of the initial 2-years intervention (i.e., 14 years after study entry).

Data on hospital admissions, involuntary in- and out-patient treatment, out-patient contacts and mortality (1992–2011) were obtained from the official Patient Administrative System, a standard system for clinical data in the health trust.

Data were cross-checked with and verified through scrutiny of the medical records. Data were assembled for each participant from the date of randomization to intervention and 14 years forward. The 12-year follow-up covered a period beginning 2 years after randomization.

### Statistics

Categorical variables were analysed using Chi-squared tests. Continuous variables were analysed using Student’s t-test. A log rank test was used to compare the survival time distribution before re-hospitalization of the two groups. The α-level was set to 0.05. SSPS version 18 was used for statistical analysis. The statistical tests for the outcome variables are listed in Table 2.

**Table 2:** Twelve year follow-up: use of psychiatric services, coercion and suicides among patients with Integrated Treatment (IT) and Treatment As Usual (TAU)

	**All (n = 45)**	**IT (n = 28)**	**TAU (n = 17)**	**Statistics**	**Chi Square -/ t-value**	**P-value**
Admitted to psychiatric wards. No. (%)	28 (62)	15 (54)	13 (77)	Chi-Square	2.36	0.125
Time to first readmission. Mean (SE)	2177 (277)	2474 (356)	1687 (414)	Log rank	2.06	0.152
Days as inpatient. Mean (SD)	385 (591)	364 (610)	420 (574)	t-test	−310	0.758
Involuntary admitted. No. (%)	23 (51)	11 (39)	12 (71)	Chi-Square	4.15	0.042*
No. of admissions as inpatient. Mean (SD)	5.0 (7.2)	4.4 (7.9)	6.0 (5.9)	t-test	-.740	0.463
Frequent users: 100–500 inpatient days. No. (%)	14 (31)	6 (21)	8 (47)	Chi-Square	3.27	0.072
Extensive users: more than 500 inpatient days. No. (%)	10 (22)	6 (21)	4 (24)	Chi-Square	.03	0.869
Psychiatric outpatient follow-up. No. (%)	44 (98)	28 (100)	16 (94)	Chi-Square	1.68	0.194
No. of psychiatric outpatient visits. Mean (SD)	107 (109)	114 (110)	96 (110)	t-test	.540	0.592
Suicides. No.	1	0	1	-	-	-
Outpatient coercion. No. (%)	14 (31)	8 (29)	6 (35)	Chi-Square	.22	0.637
Days involuntary admitted. Mean (SD)	263 (525)	265 (591)	259 (413)	t-test	.033	0.974
Days outpatient coercion. Mean (SD)	252 (511)	221 (463)	305 (594)	t-test	-.528	0,600
Total days under coercion. Mean (SD)	515 (945)	486 (962)	564 (943)	t-test	-.266	0.791

*No*. number, *SE* standard error, *SD* standard deviation, * = significant difference.

## Results

### Participants flow

In the two-year IT intervention period, five patients had reduced treatment participation and retention. Four TAU patients received very limited follow-up and psychosocial treatment. All participants were included in the assessments in the two-year intervention period.

In the long-term follow-up study complete data on 45 of the 50 patients were accessible from hospital records. Five patients had migrated from the region (two from the IT group). One patient from the control group died four years and nine months into the follow-up period. We included all data from the deceased participant in the 12-year follow-up.

Outcome measures are summarized in Table 2. There was a significant group difference in number of patients involuntary admitted to hospital during the follow-up period (p = .042, phi = .30). No other significant differences were found between the IT and TAU groups. An analysis of covariance (ANCOVA) controlling for days hospitalized 12 months preceding study entry, showed no significant difference between the IT and the TAU group in mean days hospitalized during 12 years following treatment trial. (p = .761).

In the IT group 12 were frequent users (more than 100 inpatient days during follow up period) of these six were extensive users (more than 500 inpatient days). In the TAU group 12 were frequent users (ns), four of these extensive users (Table 3). There was one death in the TAU group due to complications following suicide attempt.

**Table 3:** Inpatient days during 12 years follow-up period divided according to extent of use

**No. of inpatient days**	**No. of patients (%)**	**Total no. of inpatient days (%)**	**Percent of total inpatient days per patient**
0	17 (38)	0 (0.0)	0.0
1–100 days	4 (9)	273 (1.6)	0.4
100–500 days	14 (31)	3949 (22.8)	1.6
>500 days	10 (22)	13104 (75.6)	7.6

N = 45.

**Figure 2:**
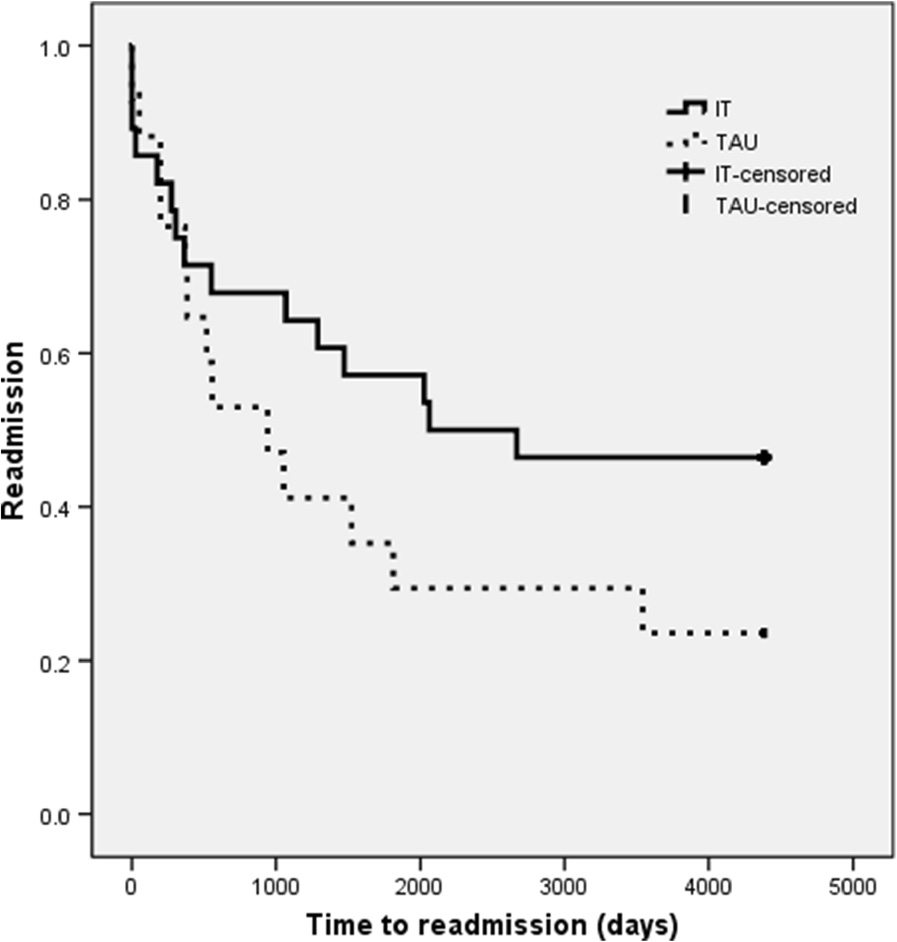
**Survival curves for time to readmission after termination of 2 years of treatment trial.** IT: Integrated treatment, TAU: treatment as usual. Log rank: P = 0.152.

A log rank test comparing the survival time distributions before readmission of the two groups showed no significant differences on time to readmission between the IT and TAU groups (Χ^2^ = 2.055, df = 1, p= > .05). A Kaplan-Meier survival curve is shown in Figure 2. The mean time (95% CI) to readmission for the IT group was 2 474 days compared to 1 687 days for the TAU group.

## Discussion

To our knowledge this is the first long term RCT follow-up (12 years) study on intensive Integrated Treatment for patients with recent-onset schizophrenia.

Significantly fewer patients receiving IT (11 of 28 patients in the IT group and 12 of 17 in the TAU group) were involuntary hospitalized. This is an important finding and should have implications in planning of services for patients with schizophrenia. There were, however, no difference in the number of days they were involuntary admitted. No significant differences were found between groups receiving integrated treatment or treatment-as-usual with respect to number of inpatient days, number of patients admitted to psychiatric wards, number of admissions to psychiatric wards, use of outpatient coercion or number of outpatient contacts over a period of 12 years. Although median time to readmission was considerably longer for the IT group, this difference did not reach statistical significance (possibly because of the sample size). Given that no effects on hospitalization were found in the first two years, these findings might not be surprising and limited conclusions can be drawn given that only 45 patients were included in the analyzes.

The findings are in line with other recent studies on 3–5 year outcomes reporting that time-limited early specialized interventions for schizophrenia do not improve outcome over time [10,11].

This lack of significant long-term effects on the use of services may be understood in several ways. Time-limited evidence- based psychosocial and pharmacological interventions do alleviate the present suffering from the illness, but may not change or hamper the long-term course of the illness [19]. It is, however, possible that there are long-term effects of such interventions, but that such effects only applies to subgroups of patients responding to elements of the interventions [5]. It is interesting that 10 of the 45 patients in this study counted for 76% of all inpatient days during the 12-year follow-up period. This group of patients with schizophrenia with extensive use of psychiatric services may represent a group which needs special attention and could potentially gain much from long term standardized care.

In the original study, IT had a beneficial impact on negative symptoms, minor psychotic episodes, and stabilizing positive symptoms [6]. In modern psychiatric care, improvements in negative symptoms might reduce the need for community services more than reduce the need for hospital admissions.

In this study the participants had recent-onset but established psychosis. The majority (87%) entered the study after their first hospitalization and had been hospitalized on average over 100 days before inclusion.

It is possible that integrated interventions at an earlier stage could have improved their long-term use of services. Such an assumption is supported by the results from the early Treatment and Intervention in Psychosis study where early detection and intervention of recent onset schizophrenia improved 10 year outcomes [20,21]. In this study the patients in both the experimental and the control group received early intervention treatment. The difference was that those in the experimental group received this treatment significantly earlier then the controls (shorter duration of untreated psychosis). Support for the effects of even earlier interventions comes from a recent meta-analysis on “risk of transition to psychosis” among persons with high risk for psychosis. This study suggests that transition into psychosis among high risk persons may be reduced by cognitive therapy and antipsychotic medications [22].

Yet another explanation for the lack of long-term effects on the use of services of the early integrated treatment demonstrated in our study is that the treatment was too time-limited. The effects of early interventions during the treatment period have been proven through multiple trials. Long-term continuous interventions may be needed if clinically significant effects are to be sustained. In the Prevention and Early Intervention Program for Psychoses in London, Ontario, long-term symptom improvements were obtained after providing the patients with five years continued, albeit less intensive, specialized treatment [23].

Involuntary admissions remain a controversial medical procedure associated with ethical dilemmas and patients with schizophrenia are among those most often involuntary admitted [24]. Research show that involuntary treatment is associated with more severe illness, reduced treatment motivation and less insight [25].

Reduction in the use of involuntary admissions is an important treatment goal. Our results suggest that long-term reduction in involuntary admissions of patients with schizophrenia is attainable by providing early integrated treatment. Forced admissions are not only a result of symptom exacerbations, but may be associated with other psychosocial variables as insight [26], violence and adherence to psychosocial treatment [27]. It is therefore possible that the group difference in forced admissions could be explained by variety of such other aspects of the illness. Components of the IT such as cognitive–behavioural family communication and problem solving skills training, individual cognitive-behavioural strategies for residual symptoms and disability have many similarities to a “crisis plan” which is a psychiatric intervention aimed specifically at reducing use of coercive measures [28]. A RCT on the use of crisis plans found a significant reduction in use of involuntary admissions [29]. This was not associated with differences in overall admissions, as is the case in our finding.

Use of in- and outpatient services is only one of many factors used as outcome measure in schizophrenia. Recovery in schizophrenia is a complex and multi-dimensional concept encompassing both objective and subjective outcome dimensions [30] and an ongoing change progress [31]. Objective outcome dimensions include symptom remission, employment, housing and relations to others while subjective outcome dimensions include appraisal of life circumstances and self-appraisal. Because of the limited scope of outcome measures in this study the possible long-term effects of IT on these multiple aspects of recovery may be hidden and not revealed.

Data from one patient in the TAU group who died 4 years and 9 months into the follow-up period were included in the statistical analyses and no statistical corrections were made to make up for the lack of data for the remaining period. As this patient died of suicide, which is the worst possible clinical outcome, it would be likely that this patient’s long-term clinical outcome would have been poor if the patient had lived. Therefore the inclusion of this patient’s data in the analyses may somewhat have influenced the results for the TAU group.

### Limitations

A sample size of 45 is too low to reveal reliable differences between the two treatment groups. Only a large sample size or a large effect size would be sufficient to test the hypothesis that the groups do not differ on number of days as in-patients or number of admittances as in-patients. P-values between 0.01 and 0.05 should be interpreted with caution, because of multiple analyses.

This follow-up RCT study is based solely on register data from psychiatric in- and outpatient services. Whether all psychiatric admissions and outpatient treatment periods were attributable to psychotic illness cannot be ascertained.

Another possible source of bias was that the researchers were not blinded or prevented from knowing which group the patients belonged to. However, because the outcome data were based upon objective information on dates and number of incidences, and because the admission data were collected for clinical and legal purposes (and not as a part of the study), the risk of information bias is considered to be minimal.

Use of in- and outpatient services may not be an optimal measure of outcome in schizophrenia and other aspects of recovery should also be considered. Clinically unstable patients and patients with substance abuse were excluded from the study and this possibly reduces the generalizability of the study.

Despite these shortcomings this is the first RCT covering a follow-up period of 12 years. This is considered to be important information for clinical practice. The long observation period and the possibility to get complete data from 90% of the participants are major strengths in the study.

### Clinical implications

The findings of this long term follow-up study, on the effects of integrated treatment for recent onset psychosis, further strengthen the implications from other recent studies that the short-term effects of early time-limited integrated treatment may not be sustainable. We found that involuntary treatment of patients with schizophrenia can possibly be prevented with early integrated treatment efforts.

Our findings must be replicated before any conclusions can be drawn about the effect of IT on long-term use of in- and outpatient services.

### Future research

RCTs on long-term effects of interventions including early identification and continuous integrated treatment for schizophrenia, focusing on different aspects of recovery, are needed.

Psychological treatments for schizophrenia as a whole are emerging and of potential importance for future studies are the effects of emerging integrative forms of psychotherapy.

It is also important to gain more knowledge about how poor and good outcome schizophrenia can be identified at an early stage of the illness in order to optimize the allocation of health resources.

## Conclusion

The intensive two-year psychosocial intervention seemed to have little long-term effects on use of in- and outpatient services. Significantly fewer patients receiving IT were involuntary hospitalized during the follow up period.

## Competing interests

The authors declare that they have no competing interests.

## Authors’ contributions

VS, GM and RWG contributed to the conception and design, the analysis and interpretation of the data. All authors drafted the article and revised it critically. All authors read and approved the version of the manuscript to be published.

## Pre-publication history

The pre-publication history for this paper can be accessed here:

http://www.biomedcentral.com/1471-244X/13/200/prepub
